# Implementation of a Self-Management Approach for Low Back Pain in a Public Health Care System

**DOI:** 10.1001/jamanetworkopen.2025.52143

**Published:** 2026-01-08

**Authors:** Ron Feldman, Tamar Pincus, Orna Reges, Alexandra Gorelik, Rachelle Buchbinder, Noa Ben Ami

**Affiliations:** 1 Department of Physiotherapy, Ariel University, Ariel, Israel; 2 Department of Physiotherapy, Central District, Maccabi Healthcare Services, Tel-Aviv, Israel; 3 School of Psychology, University of Southampton, Southampton, United Kingdom; 4 Department of Health Systems Management, Ariel University; Ariel, Israel; 5 Musculoskeletal Health and Wiser Health Care Units, School of Public Health and Preventive Medicine, Monash University, Melbourne, Australia

## Abstract

**Importance:**

Self-management is recommended first-line care for chronic low back pain (CLBP). However, implementation of structured self-management models in large public health maintenance organizations (HMO) remains scarce.

**Objective:**

To determine the feasibility of implementing the enhanced transtheoretical model intervention (ETMI) in a public HMO in Israel and to compare outcomes of patients who received ETMI-guided vs usual physiotherapy care.

**Design, Setting, and Participants:**

In this cohort study, all physiotherapists in 13 outpatient physiotherapy clinics in a public HMO were trained in ETMI in 2022. Data were extracted for all eligible patients aged 18 years and older with CLBP who attended an associated physiotherapy center between January 1, 2022, and July 31, 2023.

**Exposure:**

ETMI is a self-management approach focused on reassurance, addressing unhelpful beliefs, and encouraging recreational physical activity, delivered without passive treatments or prescribed exercises. ETMI trains physiotherapists to guide patients in managing their pain independently, helping them avoid future reliance on medical services, medication, or imaging when pain recurs.

**Main Outcomes and Measures:**

Implementation outcomes were reach (physiotherapist uptake of ETMI), adoption (number of patients who received ETMI), and fidelity. Clinical outcomes were patient-reported function (0-100), pain (0-10) and fear-avoidance beliefs (0-100), assessed at baseline and discharge.

**Results:**

A total of 128 physiotherapists (mean [SD] age, 37.5 [9.3] years; 63 [49.2%] female) were invited to attend ETMI training, 109 (85.1%) attended, and 70 (64.0%) delivered the intervention at least once. Fidelity to ETMI-guided care was good. Of 4193 patients (mean [SD] age, 56.3 [16.7] years; 2454 [58.5%] female), 711 (17.0%) received ETMI-guided care. A total of 1624 patients (39.0%) had complete data and were included in the analysis. Compared with usual care, ETMI was associated with greater improvement in function scores (mean [SD] change, 12.0 [13.7] vs 15.7 [14.1]; adjusted mean difference (aMD), 3.3; 95% CI, 1.5 to 5.1), and fear-avoidance scores (−4.4 [22.7] vs −8.9 [23.8]; aMD, −4.3; 95% CI, −1.7 to −7.0) with no differences observed for pain scores (−1.7 [2.4] vs −2.0 [2.4]; aMD, −0.0; 95% CI, −0.3 to 0.3). ETMI patients had fewer sessions (mean [SD], 5.0 [4.2] vs 6.3 [4.5]; aMD, 1.3; 95% CI, 0.6 to 1.8; *P* < .001).

**Conclusions and Relevance:**

In this cohort study, the implementation of ETMI was feasible and associated with better patient outcomes in fewer treatment sessions than usual care, although its reach was limited.

## Key Points

**Question:**

Can a structured self-management intervention, the enhanced transtheoretical model intervention (ETMI), for people with chronic low back pain (CLBP), be feasibly implemented in a public health care system, and what patient outcomes are observed?

**Findings:**

In this cohort study of 128 physiotherapists and 4193 patients, 85.1% of physiotherapists were trained and 64.0% implemented ETMI in 711 patients with CLBP. Compared with usual care, ETMI-guided care was associated with greater improvements in function and fear-avoidance beliefs and required fewer treatment sessions.

**Meaning:**

These findings suggest ETMI has the potential to improve outcomes for people with CLBP in fewer sessions, but its limited reach highlights challenges in large-scale implementation.

## Teaser

This cohort study evaluates the feasibility of implementing the enhanced transtheoretical model intervention, a self-management approach to physiotherapy, and compares outcomes of patients who received the intervention vs usual physiotherapy care.

## Introduction

Clinical guidelines for chronic low back pain (CLBP) recommend first-line management should include reassurance about the likely good long-term prognosis and assisting patients to self-manage their symptoms.^1,2,3^ Barriers to implementing these recommendations include misconceptions among clinicians about the causes and treatment of CLBP,^4,5^ limited knowledge and confidence in evidence-based approaches,^5,6^ and patient expectations about care^7,8^ Time constraints also make it challenging to deliver personalized, high-quality treatment.^9,10^ Additionally, encouraging people to be more physically active and reduce sedentary behavior remains difficult.^11^

In usual physiotherapy care for people with CLBP, treatment typically includes a combination of exercise therapy, education, manual techniques (such as mobilization or soft-tissue therapy), and adjunct modalities (eg, electrotherapy, dry needling, or heat and/or cold therapy). By contrast, the enhanced transtheoretical model intervention (ETMI) is a self-management approach for people with CLBP.^12^ Health care practitioners are trained to support patients by addressing unhelpful beliefs and providing education about natural recovery, helping them overcome barriers to self-management, and encouraging leisure-time physical activity.^12^ They are trained to use exposure to movement, accompanying patients during brisk walking sessions to demonstrate that movement is safe and not harmful. Through this process, patients gain confidence in their ability to move. By promoting active coping rather than focusing on symptom reduction, this approach helps patients reinterpret back pain as a common and self-limiting condition, thereby reducing fear avoidance and improving function.^12^ A central component of ETMI is helping patients to understand that regular physical activity is key to preventing and managing low back pain.^13^

A pragmatic clinical trial of ETMI vs usual physiotherapy care in people with CLBP (8 centers, 34 physiotherapists, and 220 CLBP participants) demonstrated that it is feasible to train physiotherapists to deliver ETMI within a short time frame, and its delivery resulted in significant improvements in function and reduced health care utilization.^12,14^ ETMI-guided care has been demonstrated to be acceptable to patients.^8^ The overall aim of this study was to investigate the feasibility of implementing ETMI-guided care for people with CLBP in 1 district (13 physiotherapy clinics) of Maccabi Healthcare Services (MHS). MHS is the second-largest public health care organization in Israel, operating across 5 districts and providing health care services to a population exceeding 2.5 million people nationwide. The primary aims were to determine the number of ETMI-trained physiotherapists who opted to use ETMI in practice (reach), the number of eligible patients who received ETMI-guided care (adoption), and the fidelity of the ETMI approach in their practice. We also assessed satisfaction with the ETMI method. Secondary aims were to explore the association between receiving ETMI-guided or usual physiotherapy care and clinical outcomes (function, pain, and fear-avoidance beliefs), and number of clinic visits. Finally, we also examined the association between depression and/or anxiety and clinical outcomes.

## Methods

### Study Design

This was a pragmatic implementation and cohort study comparing clinical outcomes of patients who received ETMI-guided vs usual physiotherapy care. It was informed by the Standards for Reporting Implementation Studies (StaRI) framework for implementation studies,^15^ and we followed the Strengthening the Reporting of Observational Studies in Epidemiology (STROBE) reporting guideline for cohort studies.^16^ The study was approved by the MHS institutional review board, which granted a full waiver of informed consent due to use of deidentified data. The study was registered at Clinicaltrials.gov on March 25, 2021 (NCT04819009).

### Participants (Clinicians and Patients)

All physiotherapists working in 13 physiotherapy clinics in the Central District of MHS, a large public health maintenance organization in Israel, with at least 1 year of experience were invited to participate in ETMI training starting in January 2022. Participation was voluntary, and no financial incentives were provided. Physiotherapists who completed the ETMI training were asked to document the use of ETMI in patient care using the ETMI code in MHS’s database.

We included all patients aged 18 years or older who had received a diagnosis of CLBP based on the *International Statistical Classification of Diseases, Eleventh Revision, Clinical Modification* classification and sought treatment at a physiotherapy center within MHS’s central district between January 1, 2022, and July 31, 2023. We excluded patients with cancer.

### Intervention: The ETMI Approach and Training

The ETMI approach is summarized in the eTable in Supplement 1 and described in detail elsewhere.^12,17^ In brief, it emphasizes the importance of listening to the patient’s story and validating their experiences, identifying and addressing misconceptions about the back problem, providing educational messages about the natural history of LBP, and providing reassurance that most patients will improve without treatment. It stresses the importance of physical activity and a healthy lifestyle, and the messages are tailored to the patient’s stage of change according to behavior change theory.^18,19^ In contrast to traditional physiotherapy care, no passive treatments or prescribed standard exercise programs are provided. Instead, the focus is on supporting the patient to do more physical activity they choose and enjoy. Patients who are hesitant about physical activity are provided with a safe exposure to brisk walking or running.

Participating physiotherapists attended 2-hour training sessions on ETMI during regular staff meetings. The first session focused on teaching the ETMI approach, while the second session allowed physiotherapists to share their experiences and challenges after using ETMI-guided care. The invitation was sent to all 13 clinics, and the managers selected 2 to 3 physiotherapists to participate in a 4-hour ETMI workshop (30 physiotherapists). This method was designed to ensure the identification and monitoring of difficulties that occurred during ETMI implementation. Following the ETMI training, participants were asked to complete a sociodemographic questionnaire and assess their satisfaction with the ETMI approach using a 5-point Likert scale, with response options ranging from very satisfied to very dissatisfied.

### Outcome Measures

#### Implementation Outcomes

We determined the proportion of physiotherapists who adopted ETMI (reach) and number of patients receiving ETMI-guided care (adoption). To assess physiotherapists’ fidelity in applying ETMI, we performed in-person observations of 14 randomly selected physiotherapists at 7 different physiotherapy clinics. Fidelity was assessed using a study-specific ETMI fidelity checklist (eAppendix in Supplement 1), developed based on the core components of the ETMI approach.^12^ The checklist included items reflecting key communication behaviors, delivery of evidence-based messages about LBP, goal setting, and promotion of physical activity. Each item was rated as performed or not performed, and overall fidelity was expressed as the proportion of ETMI components delivered as intended. Satisfaction with the ETMI method was assessed on a Likert scale (very satisfied, satisfied, neither satisfied nor dissatisfied, dissatisfied, or very dissatisfied).

#### Patient Outcomes

As part of MHS routine care, all patients are asked to complete the Focus On Therapeutic Outcomes Lumbar Computerized Adaptive Test^20^ at baseline and at discharge. This tool has been validated for use in patients with back pain seeking rehabilitation in outpatient therapy clinics and is reliable and sensitive to change.^21^ It includes self-reported function measured on a linear scale of 0 to 100, with a higher score indicating greater function; self-reported pain intensity in the last 24 hours measured on a numeric rating scale of 0 to 10, with 10 indicating severe pain; and the Physical Activity subscale of the Fear Avoidance Beliefs Questionnaire (FABQ-PA) with scores (0 to 24) transformed to a 0 to 100 scale, where higher scores indicate greater fear of movement. The Hebrew version of the tool was cross-culturally translated using standardized procedures and is widely used in clinical practice.^22^ In populations similar to ours, the minimal clinically important difference (MCID) for function has been reported to range from 3 to 9 points, depending on the baseline score (overall MCID is 5, MCID is 3 for a baseline score of >51-58 and MCID is 9 for a baseline score <44)^21^; for pain, it has been reported to be 2 points,^23^ and for FABQ-PA it has been reported to be 4 points.^24,25^

### Data Collection

We extracted data about all eligible physiotherapists from the central district in MHS, including their age, gender, years of experience, and whether they had a masters degree. Extracted patient data included gender, age, socioeconomic score (a weighted socioeconomic measure derived from economic and social data from the Israeli Central Bureau of Statistics, scored on a continuous scale from 0 to 10, with higher scores indicating higher socioeconomic position),^26^ body mass index, level of physical activity (none, 1-2 times per week, or 3 or more times per week), comorbidities, number of physiotherapy visits, use of ETMI, self-reported function, and pain and fear avoidance beliefs about physical activity at baseline and care discharge.

### Statistical Analysis

All relevant data were extracted from MHS database and exported into SPSS version 27 (IBM) for analysis. All continuous data were tested for normality using the Shapiro-Wilks test. Summary statistics were used to describe both physiotherapists and patients included in and those excluded from the study. The results were reported as means (SD) for continuous variables and as numbers (percentages) for categorical variables. Similar methods were used to describe patients treated with ETMI and those treated with usual care.

The outcomes of ETMI on changes in function, pain, and fear-avoidance beliefs from baseline to discharge among CLBP patients compared with those who received usual physiotherapy care were assessed using *t* tests for continuous data and χ^2^ tests for categorical variables. A multilevel mixed-effect linear regression model with random effects for physiotherapist identification was used to examine the outcomes of ETMI (yes or no) on changes in function, pain, and fear-avoidance beliefs while controlling for age, gender, socioeconomic score as a continuous variable, diagnosed depression and/or anxiety, and the level of each variable at baseline.

The effects of depression and/or anxiety on the outcomes of interest are well established.^27^ Therefore, we not only adjusted the main analysis for baseline depression and/or anxiety, we also examined the association between ETMI and changes in function, pain, and fear avoidance beliefs score in the subset of patients with established depression and/or anxiety using the same methods.

The level of significance was set at *P* < .05 for all tests. The analysis of secondary or descriptive outcomes was not adjusted for multiplicity and should not be used to infer definitive treatment effects. All results were interpreted with respect to both statistical significance and clinical relevance.

## Results

All 128 eligible physiotherapists (mean [SD] age, 37.5 (9.3) years; 63 [49.2%] female) agreed to participate in the ETMI training. A total of 109 (85.1%) completed the training and 19 (all from a single clinic) dropped out after the first session, citing resistance to the shift from traditional physiotherapy treatments to recommending physical activity and supporting self-management. Demographic factors were similar among those who did and did not complete ETMI training (Table 1).

**Table 1.** Sociodemographic Characteristics of Physiotherapist Participants

Variable	Participants, No. (%)				
	All physiotherapists (N = 128)^b^	Did not complete ETMI training (n = 19)	Completed ETMI training (n = 109)^a^		
			Overall	Used ETMI with at least 1 patient (n = 70)	Did not use ETMI (n = 39)
Age, mean (SD), y	37.5 (9.3)	37.6 (9.0)	37.6 (9.2)	37.8 (9.3)	37.2 (9.1)
Sex					
Female	63 (49.2)	11 (57.9)	52 (47.7)	34 (48.6)	18 (46.2)
Male	65 (50.8)	8 (42.1)	57 (52.3)	36 (51.4)	21 (53.8)
Years of experience					
<5	18 (14.1)	5 (26.3)	13 (11.9)	9 (12.9)	4 (10.3)
5-10	37 (28.9)	9 (47.4)	28 (25.7)	17 (24.3)	11 (28.2)
10-20	51 (39.8)	2 (10.5)	49 (45.0)	30 (42.8)	19 (48.7)
>20	22 (17.2)	3 (15.8)	19 (17.4)	14 (20.0)	5 (12.8)
MSc degree	63 (49.2)	11 (57.9)	52 (47.7)	34 (48.5)	18 (46.1)
Satisfaction with the ETMI method^c^					
Very satisfied	NA	NA	83 (76.1)	63 (90.0)	20 (51.3)
Satisfied	NA	NA	12 (11.0)	6 (8.5)	6 (15.3)
Neither satisfied nor dissatisfied	NA	NA	9 (8.3)	1 (1.5)	8 (20.5)
Dissatisfied	NA	NA	3 (2.8)	0	3 (7.7)
Very dissatisfied	NA	NA	2 (1.8)	0	2 (5.2)

Abbreviations: ETMI, enhanced transtheoretical model intervention; MSc, Master of Science; NA, not applicable.

^a^ Attended both ETMI sessions.

^b^ Attended the first ETMI session.

^c^ Satisfaction with the ETMI method was significantly better in the trained physiotherapists who used ETMI-guided care vs those who did not; *P* < .001.

### Implementation Outcomes

Of those who completed the ETMI training, 70 (64%) implemented the intervention: 21 (30%) used it with 10% to 20% of their CLBP patients, 28 (40%) with 20% to 40%, and 21 (30%) with more than 40%. There were no differences in terms of gender, age, years of experience, or master qualification between physiotherapists who used ETMI and those who did not. Physiotherapists who used ETMI expressed greater satisfaction with the ETMI method compared with those who did not (odds ratio, 8.55; 95% CI, 3.14-23.29; *P* < .001).

Fidelity to ETMI-guided care was good among the 14 physiotherapists who were observed. Of these, 12 sessions (85.7%) were properly documented, 9 physiotherapists (64.2%) communicated evidence-based information on LBP, 13 (92.8%) discussed healthy lifestyles, and 10 (71.4%) conveyed the essential messages.

Overall, 4303 patients with CLBP attended at least 1 physiotherapy session during the study period (Figure). One hundred and ten (2%) were excluded due to a cancer diagnosis. Of the remaining 4193 eligible patients (mean [SD] age, 56.3 [16.7] years; 2454 [58.5%] women), 711 (17%) received ETMI-guided care at least once.

**Figure 1.**
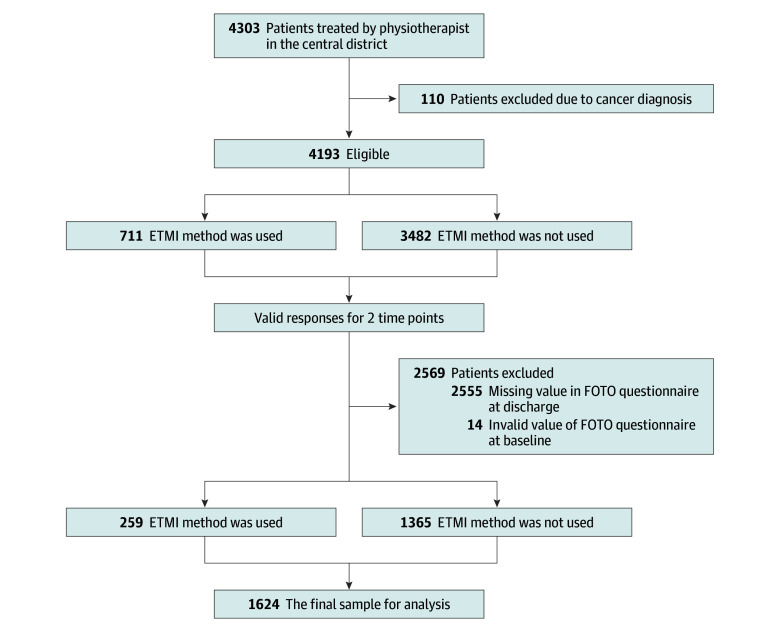
Flow Chart of Study Population Selection

Fourteen patients (0.3%) had incomplete baseline data, and 2555 (60.9%) did not attend their final treatment session and therefore did not have discharge data. The final analysis-ready dataset therefore included 1624 patients (39%), of whom 259 (15.9%) received ETMI-guided care and 1365 (84.1%) received usual physiotherapy care.

The sociodemographic and baseline clinical characteristics and referral pathways of patients according to whether or not they were included in the analysis and comparing those who received ETMI-guided care with those who did not are shown in Table 2. Overall, there were no clinically meaningful differences in baseline sociodemographic or clinical characteristics between those who did and did not receive ETMI-guided care, irrespective of whether they were included in the outcome analysis.

**Table 2.** Sociodemographic and Baseline Clinical Characteristics and Referral Pathway to Physiotherapy of Eligible Patients With Chronic Low Back Pain

Variable	Included in the analysis (n = 1624)			Excluded from the analysis (n = 2569)		
	Participants, No. (%)		*P* value	Participants, No. (%)		*P* value
	ETMI (n = 259)	No ETMI (n = 1365)		ETMI (n = 452)	No ETMI (n = 2117)	
Age, mean (SD), y	55.6 (15.8)	57.1 (16.8)	.16	54.8 (16.5)	56.6 (16.7)	.04
Socioeconomic score (0-10, 10 is high), mean (SD)	7.3 (2.0)	7.3 (2.2)	.91	7.1 (2.1)	7.2 (2.2)	.29
BMI, mean (SD)^a^	27.2 (5.3)	27.6 (5.1)	.28	28.0 (5.1)	27.9 (5.4)	.86
No. of physiotherapy visits, mean (SD)	5.0 (4.2)	6.3 (4.5)	<.001	2.9 (3.2)	3.9 (4.2)	<.001
Baseline function (0-100, 100 is a better function), mean (SD)	47.5 (13.7)	47.1 (14.0)	.70	51.4 (15.6)	46.7 (15.5)	<.001
Baseline pain score (0-10, 0 is no pain), mean (SD)	6.0 (2.5)	5.6 (2.5)	.01	5.4 (2.6)	4.3 (2.7)	<.001
Baseline fear avoidance beliefs (0-100, 100 is better), mean (SD)	45.3 (21.6)	45.2 (22.1)	.96	45.0 (21.6)	44.9 (22.6)	.96
Sex						
Female	150 (57.9)	783 (57.4)	.87	263 (58.2)	1258 (59.4)	.63
Male	109 (42.1)	582 (42.6)	.86	189 (41.8)	859 (40.6)	.67
Hypertension	71 (27.4)	434 (31.8)	.16	122 (27.0)	643 (30.4)	.15
Diabetes	23 (8.9)	187 (13.7)	.03	45 (10.0)	290 (13.7)	.03
Ischemic heart disease	13 (5.0)	127 (9.3)	.02	39 (8.6)	210 (9.9)	.40
CVA/TIA	4 (1.5)	31 (2.3)	.46	8 (1.8)	49 (2.4)	.48
Anxiety or depression	30 (11.6)	176 (12.9)	.56	74 (16.4)	312 (14.7)	.38
No. of physical activity sessions per week						
0	100 (38.6)	495 (36.2)	.76	141 (31.2)	750 (35.4)	.37
1-2	81 (31.3)	435 (31.9)		154 (34.1)	687 (32.5)	
≥3	78 (30.1)	435 (31.9)		157 (34.7)	680 (32.1)	
Referral pathway to physiotherapy						
Orthopedic specialists	68 (26.3)	229 (16.8)	.001	161 (35.6)	433(20.5)	<.001
Family physician	3 (1.2)	25 (1.8)		3 (0.7)	53 (2.5)	
Self-referral (direct access)	188 (72.6)	1111 (81.4)		288 (63.7)	1630 (77.0)	
Unknown	0	0		0	1 (<0.1)	

Abbreviations: BMI, body mass index; CVA, cerebrovascular accident; ETMI, enhanced transtheoretical model intervention; TIA, transient ischemic attack.

^a^ Calculated as weight in kilograms divided by height in meters squared.

A higher proportion of patients included in the outcome analysis were self-referred, and this was consistent across ETMI and non-ETMI groups (χ^2^_3_ = 51.8; *P* < .001). Patients who received ETMI-guided care had fewer treatment sessions than patients who received usual care, and this was also consistent across both those included and excluded from the outcome analysis (mean [SD] 5.0 [4.2] vs 6.3 [4.5]; adjusted mean difference [aMD], 1.3; 95% CI, 0.6 to 1.8; *P* < .001 and 2.9 [3.2] vs 3.9 [4.2]; aMD, 1.0; 95% CI, 0.6 to 1.3; *P* < .001, respectively).

### Patient Outcomes Analysis

Among the 1624 patients included in the outcomes analysis, those who received ETMI-guided care had greater improvements in function and fear-avoidance beliefs compared with those who received usual care (Table 3). The mean change (SD) in function score was 12.0 (13.7) with usual care and 15.7 (14.1) with ETMI-guided care (aMD, 3.3; 95% CI, 1.5 to 5.1), and the mean (SD) change in fear-avoidance scores was −4.4 (22.7) with usual care and −8.9 (23.8) with ETMI-guided care (aMD, −4.3; 95% CI, −1.7 to −7.0). Improvement in pain was similar in both treatment groups (mean [SD] change, −1.7 [2.4] with usual care and −2.0 [2.4] with ETMI-guided care; aMD, −0.0; 95% CI, −0.3 to 0.3).

**Table 3.** Baseline, Discharge, and Change in Function, Pain, and Fear Avoidance Beliefs in Patients With Chronic Low Back Pain

Variable	Mean (SD)		Adjusted mean difference (95% CI)^a^
	Usual physiotherapy care (n = 1365)	ETMI-guided care (n = 259)	
Function^b^			
Baseline	47.1 (14.0)	47.6 (13.7)	3.3 (1.5 to 5.1)
Discharge	59.1 (16.5)	63.2 (16.3)^c^	
Change	12.0 (13.7)	15.7 (14.1)^c^	
Pain^d^			
Baseline	5.6 (2.5)	6.0 (2.5)^e^	−0.0 (−0.3 to 0.3)
Discharge	3.9 (2.3)	4.1 (2.4)	
Change	−1.7 (2.4)	−2.0 (2.4)	
Fear avoidance beliefs^f^			
Baseline	45.2 (22.1)	45.3 (21.6)	−4.3 (−7.0 to −1.7)
Discharge	40.8 (21.6)	36.4 (19.8)^g^	
Change	−4.4 (22.7)	−8.9 (23.8)^g^	

Abbreviation: ETMI, enhanced transtheoretical model intervention.

^a^ Model was adjusted for age, sex, socioeconomic status, anxiety/depression, and level at baseline.

^b^ Range 0 to 100; higher score indicates better function.

^c^ *P* < .001.

^d^ Range 0 to 10; lower score indicates less pain.

^e^ *P* < .05.

^f^ Range 0 to 100; lower score indicates fewer fear avoidance beliefs.

^g^ *P* < .01.

For the subset of patients with depression and/or anxiety at baseline (206 patients), those who received ETMI-guided care (30 patients) had greater improvement in function at discharge compared with those who received usual care (176 patients). The mean (SD) change was 9.7 (16.8) with usual care and 16.8 (14.2) with ETMI-guided care (aMD, 5.8; 95% CI, 0.6 to 10.9). Improvements in pain and fear avoidance were similar in both groups (Table 4).

**Table 4.** Baseline, Discharge, and Change in Function, Pain, and Fear Avoidance Beliefs in Patients With Chronic Low Back Pain With Depression and/or Anxiety

Variable	Mean (SD)		Adjusted mean difference (95% CI)^a^
	Usual physiotherapy care (n = 176)	ETMI-guided care (n = 30)	
Function^b^			
Baseline	45.9 (13.2)	40.6 (13.8)^c^	5.8 (0.6 to 10.9)
Discharge	55.6 (15.5)	57.4 (17.4)	
Change	9.7 (16.8)	16.8 (14.2)^d^	
Pain^e^			
Baseline	5.0 (2.5)	5.2 (2.7)	−0.5 (−1.3 to 0.3)
Discharge	3.6 (2.2)	3.0 (2.7)	
Change	−1.5 (2.4)	−2.1 (2.8)	
Fear avoidance beliefs^f^			
Baseline	44.6 (22.7)	49.3 (23.9)	−5.9 (−13.9 to 2.2)
Discharge	42.1 (22.2)	37.7 (20.5)	
Change	−2.6 (24.4)	−11.6 (28.5)	

Abbreviation: ETMI, enhanced transtheoretical model intervention.

^a^ Model was adjusted for age, sex, socioeconomic status, anxiety/depression, and level of the variable of interest at baseline.

^b^ Range 0 to 100; higher score indicates better function.

^c^ *P* < .05.

^d^ *P* < .001.

^e^ Range 0 to 10; lower score indicates less pain.

^f^ Range 0 to 100; lower score indicates less fear avoidance beliefs.

## Discussion

We have demonstrated the feasibility of training physiotherapists in the ETMI approach and implementing it with fidelity into ETMI-guided care for patients with CLBP in a large public health care setting in Israel. Our findings showed that, compared with usual care, ETMI-guided care was associated with better outcomes in function and fear-avoidance beliefs and this was achieved with fewer treatment sessions. These clinical practice data are consistent with the results of a previous pragmatic clinical trial.^12,14^ Similar outcomes were also observed among the subset of patients with depression and/or anxiety, although only the difference in function was significant.

The improved function and fear-avoidance of physical activity beliefs with ETMI-guided care may reflect the behavioral focus of ETMI. The intervention targets patients’ beliefs and activity patterns rather than directly addressing pain symptoms, which may also explain a lack of difference in improvement in pain between the treatment groups.

While almost two-thirds of physiotherapists who completed training applied ETMI-guided care to at least 1 patient, ETMI-guided care was only provided to 17% of patients with CLBP overall. While this may indicate that 2 education sessions are insufficient to change practice, another explanation for the low overall uptake of the intervention is that physiotherapists may find it difficult to transition from actively treating people with CLBP to simply supporting their self-management. Previous qualitative studies with physiotherapists introduced to ETMI suggest that some see the approach as threatening to their professional identity.^28,29^ The transition from a clinician-managed approach to back pain that focuses on specific diagnoses, such as weak core muscles or poor posture, and treatments involving targeted exercises, passive modalities, and/or specialized techniques, toward a model that prioritizes patient self-management may be challenging for physiotherapists. Many are accustomed to a fixing mindset, making it difficult to shift toward empowering patients to manage their own recovery.

A previous study that determined the perceptions of patients with CLBP treated with ETMI approach found that patients had no problem with the fact that they did not receive passive treatment.^7,8^ They highlighted their need for reassurance and encouragement and perceived ETMI as a practical tool for self-management.

We found that a higher proportion of patients who had self-referred for care completed their final visit, indicating they may have had higher levels of engagement in their rehabilitation. However, there was no difference in the proportion of patients who failed to attend their final visit between those who were and were not treated with ETMI, suggesting that the type of care they received did not influence their decision to attend.

### Strengths and Limitations

Our study had a pragmatic design with high ecological validity. The results are more likely a reflection of the true uptake of ETMI-guided care in clinical practice. Physiotherapists trained in the ETMI method may also have incorporated some ETMI features into their usual physiotherapy care, which may have underestimated the benefits of the ETMI approach. In addition, ETMI exposure was based on physiotherapists’ coding in the electronic medical record. Some ETMI elements may have been applied without formal coding, which could also have reduced the observed differences between groups.

Fidelity of ETMI delivery was assessed in a relatively small sample of physiotherapists (approximately 20%). While our observations provided useful insights, they may not fully represent the practices of all physiotherapists who delivered ETMI.

Due to the extent and uncertain mechanism of missing discharge data, multiple imputation was not performed, as it would likely violate the missing-at-random assumption. Consequently, the results are based on observed data from completers and may not be generalizable to all patients who received care. Additionally, the small number of participants with depression and/or anxiety limits the interpretation of our subgroup findings, which should be considered exploratory.

## Conclusions

This cohort study found that the implementation of the ETMI approach within a large public health care system was feasible and associated with improved patient outcomes with fewer physiotherapy sessions and minimal practitioner training. Although the reach was limited, these findings highlight the potential of scalable, self-management–based interventions to enhance care efficiency and reduce resource use for people with CLBP. Wider adoption could contribute to more sustainable musculoskeletal care provided that implementation barriers are systematically addressed.

## Notes

Supplement 1. eTable. Detailed Content of ETMI Training eAppendix. ETMI Fidelity Checklist

Supplement 2. Data Sharing Statement
