# Supplementary material for: Implementation of a Self-Management Approach for Low Back Pain in a Public Health Care System
Source: JAMA Netw Open. 2026 Jan 8;9(1):e2552143. doi: 10.1001/jamanetworkopen.2025.52143 (PMC12784229; doi:10.1001/jamanetworkopen.2025.52143)
Supplement: Supplement 1. — eTable. Detailed Content of ETMI Training eAppendix. ETMI Fidelity Checklist [file jamanetwopen-e2552143-s001.pdf]

Supplemental Online Content

Feldman R, Pincus T, Reges O, Gorelik A, Buchbinder R, Ben Ami N. Implementation of a self-management approach for low back pain in a public health care system. *JAMA Netw Open*. 2026;9(1):e2552143. doi:10.1001/jamanetworkopen.2025.52143

eTable. Detailed Content of ETMI Training

eAppendix. ETMI Fidelity Checklist

This supplemental material has been provided by the authors to give readers additional information about their work.

© 2025 Feldman R et al. *JAMA Network Open*.

eTable. Detailed Content of ETMI Training

Training sessions	List activities and content
**Session 1:**<br>Two hours of face-to-face ETMI education during regular staff meetings in 13 physiotherapy clinics (128 physiotherapists)	Introduced a synthesis of international clinical guidelines for treating LBP:<br>*The burden of LBP is amplified by an epidemic of poor care, and disability may be an iatrogenic disorder<br>*Imaging, medicines, and surgery have a limited role in most patients with LBP<br>*Screening for red flags and considering psychosocial factors<br>Then, we discussed the principles and components of ETMI and how to convey Messages:<br>*The goal of ETMI is to assist patients in self-managing their symptoms<br>**Reduce the perceived threat of back pain through normalizing messages:**<br>*LBP is normal - 90% of people will have it in their lifetime - it is not dangerous<br>*Most LBP gets better within days, weeks, or months without treatment<br>*Back pain flare-ups are common; 70% of people will have them every year<br>*Pain can occur from awkward movements, muscle spasms, fatigue, and stress<br>*Typically, the pain lasts a day or two; continue with your life, and it usually passes quickly<br>*We are likely to suffer more when we worry about the pain, focus on it, or avoid movements<br>*It's like the flu: For most people, a day or two of fever and pain, and then they recover over a week or so<br>**Eliciting patients' beliefs and misconceptions about back pain. Responding with clear messages:**<br>*There are no perfect postures, correct sitting, or lifting<br>*Core exercises are no more effective than any other exercise<br>*Many types of physical activity can help; do what you enjoy<br>*LBP is rarely caused by damaged spinal structure, so you don't need imaging<br>*Most drugs have limited effectiveness and have side effects<br>*You can manage your back pain alone. Medical treatment is not needed<br>**Engaging patients in self-management and Empowering Control:**<br>*The most helpful things for managing your back pain are keeping your body strong and flexible, healthy sleep, and a healthy lifestyle<br>*Intensive physical activity is not necessary; even daily walking is excellent<br>*The important thing for you is to learn how to manage the next episode by yourself. You can do it!<br>**How to convey messages:**<br>*Patient-centered care<br>*Building a therapeutic alliance, listening to the patient's story and validating their experiences<br>*Listen to patients' preferences and encourage them to keep doing it<br>*Encouraging physical activity chosen by the patients rather than correcting movement and specific exercise<br>*Don't lecture and persuade (it doesn't work)<br>*Pay attention to their self-efficacy and the stage of readiness (what they think they can do and what they can't) and support the patient's wishes<br>* For patients who show hesitancy or worry about physical activity, provide safe exposure to brisk walking or running
**Session 2:** Two hours of ETMI training after using the ETMI approach during regular staff meetings in all 13 physiotherapy clinics (109 physiotherapists)	The second session was scheduled after the physiotherapists had attempted to use ETMI. We allotted time for them to ask questions, discuss barriers, and share difficulties they encountered with the ETMI approach. Additionally, we included role-playing examples to demonstrate its application
**Workshop:** Four-hour ETMI workshop for representatives from all 13 physiotherapy clinics (30 physiotherapists)	We discussed LBP evidence-based practice and ETMI principles again. We used videotaped interviews with patients who suffer from back pain and receive treatment according to the ETMI approach, and role-play by the attendees. We discussed the major misconceptions of physiotherapists about weak core muscles, bad postures, correcting movement, passive management, and a deeper understanding of the Transtheoretical model and Motivational interviewing. We reviewed the ETMI qualitative research, which highlights the difficulties of physiotherapists changing their usual practices.

ETMI- Enhanced Transtheoretical Model Intervention; LBP- Low Back Pain.

© 2025 Feldman R et al. *JAMA Network Open*.

eAppendix. ETMI Fidelity Checklist

**Fidelity checklist**	Clinician 1	Clinician 2	Clinician 3	Clinician 4	Clinician 5
ETMI's code documentation					
Physiotherapists used communication skills:<br>1. Active listening<br>2. Reflection<br>3. Validation<br>4. Summary					
Physiotherapists spoke according to the stage of change with their patients					
Physiotherapists communicated evidence-based information on LBP to their patients:<br>1. LBP is common and frustrating.<br>2. The prognosis is good.<br>3. Pain does not always mean damage.<br>4. Bed rest should be avoided. Rather, pace your activities at home and work.<br>5. Most cases improve without treatment within days, weeks or months.<br>6. Self-management strategies are recommended.<br>7. Did not offer non-evidence-based treatment modalities (i.e., dry needling, massage, traction, Shockwave, Orthoses, and braces)<br>8. Identify and address misconceptions about back pain					
Physiotherapists discussed healthy lifestyle and well-being:<br>1. Adequate sleep<br>2. Healthy nutrition<br>3. Smoking					
Physiotherapists used three mandatory messages:<br>1. "Physical activity is the only thing that will help your back pain over time."<br>2. "It's easy to reduce your pain now; it’s the natural recovery, but the important thing is to manage the next episode."<br>3. "Your body must be strong and flexible."					
Physiotherapists discussed the role of physical activity with their patients.					
Physiotherapists used exposure to brisk walking (not mandatory)					
**ETMI informational kit used**					
Physiotherapists referred to infographic					
Physiotherapists referred to leaflet					
Physiotherapists referred to postcards with gentle back mobility options					
Physiotherapists referred to short-animated movie clips					

ETMI- Enhanced Transtheoretical Model Intervention; LBP- Low Back Pain.

© 2025 Feldman R et al. *JAMA Network Open*.
