# Supplementary material for: Implementation of a Self-Management Approach for Low Back Pain in a Public Health Care System
Source: JAMA Netw Open. 2026 Jan 8;9(1):e2552143. doi: 10.1001/jamanetworkopen.2025.52143 (PMC12784229; doi:10.1001/jamanetworkopen.2025.52143)
Supplement: Supplement 2. — Data Sharing Statement [file jamanetwopen-e2552143-s002.pdf]

Data Sharing Statement

Feldman. Implementation of a Self-Management Approach (ETMI) for Low Back Pain in a Public Health Care System. *JAMA Netw Open*. Published January 06, 2026.
doi:10.1001/jamanetworkopen.2025.52143

Data
Data available: No
